# Transitioning from acute to chronic pain: a simulation study of trajectories of low back pain

**DOI:** 10.1186/s12967-019-2030-0

**Published:** 2019-09-06

**Authors:** Jianzhong Su, Ying Du, Kelley Bevers, Pengcheng Xiao, John Licciardone, Marco Brotto, Robert J. Gatchel

**Affiliations:** 1 Department of Mathematics, University of Texas at Arlington, Arlington, USA; 2 Department of Mathematics, East China University of Science and Technology, Shanghai, China; 3 Department of Psychology, University of Texas at Arlington, Arlington, USA; 4 Department of Mathematics, Kennesaw State University, 1100 South Marietta Pkwy, Marietta, GA 30060 USA; 5 Department of Family Medicine, UNT Health Science Center, Fort Worth, USA; 6 College of Nursing and Health Innovation, University of Texas at Arlington, Arlington, USA

**Keywords:** Low back pain, Chronic and acute pains, Pain trajectories, HPA axis, Ordinary differential equation system, Computer simulation

## Abstract

**Background:**

Identifying how pain transitions from acute to chronic is critical in designing effective prevention and management techniques for patients’ well-being, physically, psychosocially, and financially. There is an increasingly pressing need for a quantitative and predictive method to evaluate how low back pain trajectories are classified and, subsequently, how we can more effectively intervene during these progression stages.

**Methods:**

In order to better understand pain mechanisms, we investigated, using computational modeling, how best to describe pain trajectories by developing a platform by which we studied the transition of acute chronic pain.

**Results:**

The present study uses a computational neuroscience-based method to conduct such trajectory research, motivated by the use of hypothalamic–pituitary–adrenal (HPA) axis activity-history over a time-period as a way to mimic pain trajectories. A numerical simulation study is presented as a “*proof of concept*” for this modeling approach.

**Conclusions:**

This model and its simulation results have highlighted the feasibility and the potential of developing such a broader model for patient evaluations.

## Background

This paper is a continuation of an earlier study of the same subject [16], where we focused on the transition of acute pain to chronic pain. As we mentioned, the need to distinguish common and reproducible pain trajectories in the population is of great importance. The 2011 Institute of Medicine (IOM) Report was released in response to the rising pain costs and prevalence, and sparked formation of a number of interdisciplinary teams and strategies to address the pain epidemic and determine areas of concern. Understanding and defining pain trajectories was one such area in which the knowledge was still currently lacking. As previously discussed in Gatchel et al. [16], identifying how pain transitions from acute to chronic is critical in designing effective prevention and management techniques for patients’ well–being, physically, psychosocially, and financially. Also, in our previous paper [16], we reviewed the various trajectories that have been delineated by different clinical research groups, which suggested the need for a comprehensive model for understanding them. We proposed that focusing this research on low back pain (LBP) is advantageous because LBP is the most prevalent form of musculoskeletal pain, and amasses billions of dollars in associated costs each year [16, 22]. Indeed, LBP presents an opportunity to observe all stages of pain (acute, subacute, chronic), as well as distinguishing different trajectories within this group.

Kongsted et al. [27] summarized ten (10) studies of LBP trajactories over a ten-year period from 2006 to 2015 [2, 3, 7, 8, 10, 13, 14, 23, 26, 36, 51] over research on adult patients of 10 cohorts by LBP trajectory methodology. In these studies, participants with a main complaint of LBP were followed from 3 to 12 months with data collection at four (4) to fifty-two (52) time-points. Outcome measures were LBP intensity, LBP frequency (number of LBP days per week) and activity limitation. Trajectory patterns were identified using either hierarchical cluster analysis, latent class analysis, or latent class growth analysis. From two (2) to twelve (12) discrete LBP trajectory patterns have been identified in these published studies. They suggested that trajectory differentiation between acute and chronic LBP is overly simplistic, and the next step is to shift from this paradigm to one that focuses on trajectories over time. Our proposed approach to modeling the time trajectory is well aligned with this new goal to build upon these earlier pain trajectory studies.

As is well known, pain pathways include multiple brain regions, and the connection among the anterior cingulate cortex, the parieto-insular cortex, the thalamus, as well as the amygdala. Several neuronal-processing mechanisms of pain signals have been proposed, although their temporal behavior, especially during an extended period of several weeks or months, is still not fully understood. It has been known that different brain areas are involved in neuronal electric and chemical activities in response to pain, and form a distributed pain-processing network, mostly centered on the somatosensory cortex and the thalamic axis, closely associated with the pain signals and/or pain-induced stress. While a full study of pain and its transition between general acute and chronic pain is beyond our reach at this point, due to the complexity of the brain network and brain activities, LBP involves relatively isolated areas of the brain, and has been attributed to a correlation to HPA-axis activity [18, 20]. Although the sequence of brain-activity events leading to LBP can still be complex or even convoluted, it is generally agreed that the principle regions of the pain response are localized in the paraventricular nucleus of the hypothalamus, the anterior lobe of the pituitary gland, and the adrenal gland, commonly referred to as the HPA axis [45]. The HPA axis plays an important role in balancing hormonal levels for the brain, and generates high concentrations of hormones in response to pain (considered as a form of stress), which leads to many “downstream” changes [4]. A number of measurement data, such as patient self-reported trajectory data, EEG data, and cortisol level changes, can be used for the quantitative study of the HPA axis, which is the primary neuronal responding mechanism for stress and pain. From the quantitative measurements, we can utilize mechanism-based computational models to predict the temporal behavior of hormone concentrations and infer trends in pain trajectories.

Along with cortisol, adrenocorticotropin (ACTH) is one of the major hormones secreted by the HPA in the anterior pituitary region in response to severe pain and other stressors [31, 63]. Tennant et al. [53] measured ACTH serum levels in fifty-five (55) severe, chronic pain patients. This study supports other reports that pituitary–adrenal function may be altered during uncontrolled pain period and after return to normal when pain control is achieved [20, 50]. Corticotropin-releasing factor (CRF) is also released from the hypothalamus and in widespread areas of the brain following the stress or pain episode. Lariviere and Melzack [30] presented evidence that CRF can act at all levels of the neuraxis to produce analgesia; inflammation must be present for local CRF to evoke analgesia and the analgesic effects of CRF presented significant specificity for prolonged pain. The similar mechanism is also studied in human subjects [6]. Overall, pain results in a hyperarousal of the hypothalamic–pituitary–adrenal system which results in elevated serum hormone levels such as adrenocorticotropin, cortisol, and pregnenolone [52].

The current methodology of measuring hormones in the hypothalamic pituitary adrenal (HPA) axis was reviewed recently by Yeo et al. [62]. Different types of dexamethasone suppression testing are compared and described in detail in [61, 62]. Common serum cortisol measurements are performed by Mass Spectrometry, Beckman Coulter, Roche Diagnostics, Siemens ADVIA Centaur, Siemens Immulite, Abbott Architect, Vitros, Tosoh Bioscience AIA-PACK Test Cups, and urinary cortisol measurements are by Mass Spectrometry, Beckman Coulter, Roche Diagnostics, Siemens ADVIA Centaur, Abbott Architect, Vitros. Salivary cortisol is measured by Mass Spectrometry, Roche. ACTH measurements however have been less common, and they can be influenced by patients with cortisol producing adrenal adenomas or patients taking exogenous steroids. The preferred specimen for ACTH is plasma, and the measurements are done by Siemens Immulite and Roche. Urine measurement can also be done [20]. CRF (or Corticotropin-releasing Hormone, CRH)—measurement is not common. Although it is known that high levels CRH are associated with high levels of CRH binding protein, few data is available in setting up a reliable measurement protocol in this area. The evaluations of the HPA axis functions are usually through biochemical measurements, imaging studies can complement the hormonal evaluations, providing valuable information for prognosis and management. Pituitary T2 MRI, Pituitary CT scan have been used in combination with biochemical measurement [54], and Adrenal CT scan, MRI have been used to adrenal measurements in Cushing syndrome [42]. In summary, cortisol measurements remain a superior method for HPA function evaluations for convenience and reliability. In particular, the modeling work proposed here is based on continuous time data and cortisol measurements using electrochemical impedance sensor [56] provides a platform for data acquisition.

Computational neuroscience-modeling techniques (using systems of ordinary differential equations) have been utilized to develop a reliable predictive outcome model for HPA-related issues, such as ultradian and circadian patterns in normal, depressed, post-traumatic stress disorder states, and their comorbid pain [1, 47, 49, 59]. In Prince et al. [40], the authors modeled acute pain from a gating mechanism point of view, which assumes the flow of inputs through the spinal cord to the brain as a major contributor for the pain experience, and constructed a biologically plausible mathematical model. Research efforts in the past have been also focused on developing mechanistic models for stress and pain, using differential equations and computational simulation of HPA-axis activities [43]. They found similar quantitative trajectories as LBP. Therefore, one can then use computational simulation and bifurcation analysis to study the complex biological processes that are involved, complementary to experimental work. A bifurcation study is a computational investigation of trajectory patterns in a system’s parametric space, and is a powerful tool useful for investigating possible pathways of the HPA network accounting for variations in neural receptors, synaptic plasticity, as well as other factors such as neuron-conductivity degeneration. Indeed the HPA-axis process and its abnormality are highly associated with the pain and stress of muscular movement involved in LBP. The present proposed predictive-outcome model is a first step in our efforts to understand the acute/chronic pain transition mechanism and a *proof of concept* to show the feasibility of applying the computational model to pain transition, which is a high-impact medical research issue.

## Methods

### Queries

In a clinical study of 131 persons with chronic widespread pain, 267 ‘at risk’ and 56 controls [34] with abnormal HPA-axis function were associated with a high rate of fibromyalgia, a syndrome characterized by chronic widespread body pain. These results confirmed the hypothesis that the psychosocial-stress caused by pain, as well as the pain, are linked to altered HPA-axis functioning. Increased cortisol levels due to alterations in the regulation of the HPA axis at young age are also believed to be one mediating mechanism for several adult conditions, including metabolic syndrome, cardiovascular disease, and psychiatric disorders [34]. Infants born after significant exposure to stressful conditions are often small for gestational age (SGA), based on standardized growth norms. A study of 37 participants, including infants of gestational age ranging from 34 to 41 weeks, suggests SGA neonates have blunted HPA axis responses to stressors [37]. These findings are consistent with animal models showing that adverse intrauterine conditions can result in blunted cortisol responses to acute stressors, and may provide a mechanism for adult susceptibility to disease through HPA axis abnormality. The importance of the hormonal response system to stress through the HPA axis is also seen in other clinical studies, particularly stress hormones known as glucocorticoids (and primarily cortisol) that are key factors in patient alcohol dependence [48].

There is growing evidence that relative hypocortisolism, as a marker of stress-induced HPA axis dysfunction, may increase vulnerability to pain and chronic pain disorders [17–19, 28, 29, 35, 58]. HPA activities, especially cortisol trajectory, are also a useful tool in longitudinal cohort studies of pain. In a study by Paananen et al. [38], for example, their sample group included 805 participants from the *Western Australian Pregnancy Cohort (Raine) Study,* who participated in the Trier Social Stress Test (TSST) at age 18-years. The number of pain sites, pain duration, pain intensity and pain frequency were assessed at age 22 in order to measure severity of musculoskeletal (MS) pain. An abnormal HPA axis response to psychosocial stress at 18-years of age was shown to be associated with MS pain alone, and MS pain combined with increased pain sensitivity at 22-years of age.

More recent evidence also suggests that in a disordered stress-system pathway, the HPA axis activity may be responsible for the genesis and maintenance of long-term sensory and emotional problems that lead to post-traumatic pain and disability. In a study by [57], the authors used hair-cortisol and hair-normalized salivary cortisol as biomarkers of distress following traumatic injuries of whiplash or distal radius fractures. Small sample results indicated that the cortisol-waking response may become a useful biomarker of current distress as measured using the *Pain Catastrophizing Scale* [57], especially when it is normalized to three-month hair cortisol. The hair-normalized cortisol waking-response also had predictive capacity, correlating with three-month self-reported disability. Tomas et al. [55] also provided a review of HPA axis dysfunction in chronic fatigue syndrome patients. The study included evidence of enhanced corticosteroid-induced negative feedback, basal hypocortisolism, attenuated diurnal variation, and a reduced responsivity to challenge. A putative causal role for a genetic profile, childhood trauma, and oxidative stress were considered. In addition, gender was also determined as a factor, in addition to an increased frequency of HPA axis dysregulation in females. The mechanisms of the HPA appears to affect LBP by following similar pathways as chronic fatigue syndrome. Abnormal cortisol concentrations reflect differences in the biological mediation of the stress response, or may be consequent on the differential nature/magnitude of the stressor engendered by the LBP. For example, in the chronic fatigue study, an attenuated diurnal variation has also been shown [39, 55], particularly with a loss of the morning-peak of ACTH or cortisol, while challenge studies often, but not invariably, show a diminished HPA axis responsivity. This has been assessed using ACTH, cortisol, and/or 11-deoxycortisol response to pharmacological challenge employing, for example, dexamethasone combined with corticotropin-releasing hormone (CRH), insulin, inflammatory cytokines, metyrapone, a psychosocial-challenge (e.g., using the Trier Social Stress Test), and to a physiological-challenge (such as a wakening; Tomas et al. [55]).

In the proposed model, we focus on the cortisol-level as a biomarker for pain trajectory for the continuation study over the time. There are a few current in vivo techniques that allow us to monitor the cortisol-level continuously for a long duration of time. For example, [56] used an electrochemical impedance sensor to measure cortisol concentration in the interstitial fluid of a human subject. The interstitial fluid is extracted by means of vacuum pressure from micropores created on the stratum corneum layer of the skin. Other measurements of the cortisol such as the saliva sampling protocol [46], and electrochemical immunosensing platforms that are well-known for their sensitive and selective detection of cortisol in biofluids [44], have also developed as tools for continuous in vivo measurements. They will guide predictive modeling development. The recent nanosheet technology [24] also allows monitoring cortisol in low-volume perspired human sweat (cortisol dynamic range from 1 to 500 ng/mL with a limit of detection of 1 ng/mL) using electrochemical impedance spectroscopy.

In this study, we will focus on the longitudinal study of LBP, in particular the transition from acute pain to chronic pain. The HPA activity history over time is of high interest. Our modeling strategy calls for a coupled system of ordinary differential equations to represent the network of brain regions along the HPA axis, and its cortisol and adrenaline production. Specifically relating to a trajectory modeling study in LBP, we focus on outcome measures such as LBP intensity, LBP frequency (number of LBP days per week), but will ignore activity-limitation which will be a subject of a future study. Trajectory patterns in a group of experimental subjects were identified using Hierarchical Cluster Analysis, Latent Class Analysis, or Latent Class Growth Analysis [27].

### Modeling consideration

Following Sriram et al. [47], in developing the mathematical model of the cortisol molecular network for the HPA, we have made two assumptions: (i) The first order dilution-rate due to the transport of hormones and autonomous degradation are considered together. Apart from dilution/autonomous degradation, Michaelis–Menten kinetics are separately considered for the degradation of the hormones, and hormone complexes, within each specific region of the brain (hypothalamus, pituitary and adrenal); and (ii) A sufficient number of molecules is present for the reactions to take place, using continuum kinetics so that stochastic fluctuations (internal noise) are minimal.

### Hypothalamic–pituitary–adrenal (HPA) axis

**Fig. 1:**
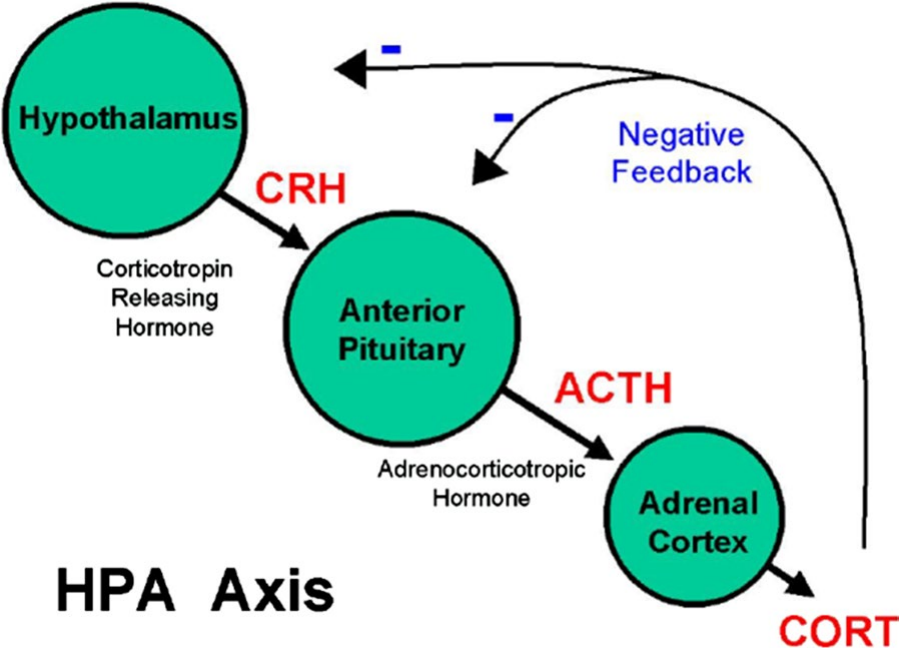
A Diagram of Hypothalamic–Pituitary–Adrenal (HPA) axis. In response to stress, the hypothalamus releases CRH, which activates the pituitary which secretes ACTH. ACTH stimulates the adrenal to secrete glucocorticoid. Glucocorticoid performs a negative feedback on the secretion of CRH and ACTH

The anatomical structure that mediates the stress response is mainly in the central nervous system (CNS) and its peripheral organs. The principle-effectors of the stress-response are localized in the paraventricular nucleus of the hypothalamus, the anterior lobe of the pituitary gland, and the adrenal gland. This collection of brain areas is commonly referred to as the Hypothalamic–pituitary–adrenal (HPA) axis [45]. The HPA axis plays an important role in balancing hormonal levels for the brain, and generate high concentrations of hormones in response to stress, which leads to “downstream changes” [43]. In response to stress over a period of time, the paraventricular nucleus of hypothalamus, which contains neuroendocrine neurons, releases corticotropin-releasing hormone (CRH). The anterior lobe of the pituitary gland is stimulated by CRH to secrete adrenal corticotrophic hormone (ACTH). The adrenal cortex then produces cortisol hormones in response to stimulation by ACTH. Cortisol is a major stress-related hormone, and has effects on many tissues in the body, particularly in the brain. In the brain, cortisol acts on two types of receptors: mineralocorticoid receptors and glucocorticoid receptors [4, 9, 41]. For “down regulating” CRH, it is known that cortisol inhibits the secretion of CRH through the glucocorticoid-receptors complex [11]. With a strong affinity to the glucocorticoid-receptors complex, cortisol, in turn, acts on the hypothalamus and pituitary in a negative feedback cycle to “down regulate” the production of cortisol [47]. Figure 1 illustrates the conceptual connection of the different brain areas in this process. Based on the biophysical relation, we established the underlying nonlinear ordinary differential equations for the cortisol network, as presented in Tables 1, 2, 3. Table 1 provides the mathematical model as in a system of ordinary differential equations. Table 2 provides the biological meaning of the parameters. A set of HPA parameters for LBP-modeling is provided in Table 3 for LBP modeling study. The model described in Table 1 is constructed by the chemical kinetics relations shown in Fig. 1. The four brain areas have excitatory or inhibitory inputs to other areas through mostly hormonal chemical kinetics relations and synaptic couplings [47]. The coefficients [47] are either from: the scientific literature; obtained by other researchers through direct measurement; or are best estimates to match experimental data through global optimization [60].

**Table 1:** Hypothalamic pituitary adrenocortical (HPA) axis model

$$ \frac{d[CRH]}{dt} = k_{stress} \frac{{K_{i}^{n2} }}{{K_{i}^{n2} + [GR]^{n2} - V_{S3} \frac{[CRH]}{{K_{m1} + [CRH]}}}}, $$	(1)
$$ \frac{d[ACTH]}{dt} = K_{p2} [CRH]\frac{{K_{i}^{n2} }}{{K_{i}^{n2} + [GR]^{n2} }} - V_{S4} \frac{[ACTH]}{{K_{m2} + [ACTH]}} - K_{d2} [ACTH], $$	(2)
$$ \frac{d[CORT]}{dt} = K_{P3} [ACTH] - V_{S5} \frac{[CORT]}{{K_{m3} + [CORT]}} - K_{d3} [CORT], $$	(3)
$$ \frac{d[GR]}{dt} = K_{b} [CORT]([G_{tot} ] - [GR]) + V_{S2} \frac{{[GR]^{n1} }}{{K1^{n1} + [GR]^{n1} }} - K_{d5} [GR] $$	(4)

**Table 2:** A list of model variables

The cortisol dynamics can be simulated in the HPA model by solving the coupled differential equations as proposed in Sriram et al. [47], where the variables and parameters are listed as follows:	
CRH	Corticotrophin-releasing hormone
ACTH	Adreno-corticotrophin hormone
CORT	Cortisol
GR	Glucocorticoid receptor complex
Gtot	Total glucocorticoid receptor
Kstress	Stress or pain induced parameter
Ki	Inhibition constant that regulates the strength of the negative feedback loop
Vs3, Vs4, Vs5	Rates at which the hormones CRH, ACTH and CORT are degraded enzymatically through saturation kinetics
Km1, Km2, Km3	Michaelis constant
Kd1, Kd2, Kd3, Kd5	Autonomous degradation constants
Kp2, Kp3, Kb	Rates of production of ACTH,CORT and GR
n1, n2	Hill constants
K1	Activation constant

**Table 3:** The model-kinetic parameters

Constants	Optimized values
Kstress	10.1 µg days
Ki	1.51 µg days
Vs3	3.25 µg days
Vs4	15 µg days
Vs5	0.00535 µg days
Km1	1.74 µg days
Km2	0.112 µg days
Km3	0.0768 µg days
Kd1	0.00379
Kd2	0.00916
Kd3	0.356
Kd5	0.0854
Kp2	8.3
Kp3	0.945
kb	0.0202
n1	5.43
n2	5.10
K1	0.645 µg days
Gtot	3.28 µg

### Computer simulation technique

We simulated the time-series data of cortisol level during a period of 100 days, based on a computational HPA-axis model, which is described in Tables 1 and 3. MATLAB solver ode45 (https://www.mathworks.com/help/matlab/ref/ode45.html) was used for its accuracy and relatively fast speed in computing this model. It should be pointed out that, in our *preliminary simulation study* to demonstrate how mathematical modeling techniques can be used to develop a better understanding of these trajectory results, it was not our intention to explore all possible scenarios.

## Results

### Preliminary simulation study

In this section, we present some examples based on the cortisol-dynamics model reported by [47], with parameters modified to fit into LBP problems. We then conducted a series of modeling pain-trajectories, based on cortisol that revealed patterns that resemble the pain trajectory patterns reported by Kongsted et al. [26]. Because cortisol secretion can be monitored in real-time, its time trajectory provides insight for studying LBP due to the inherent structure of the HPA in the brain hormone system under stress. The cortisol-level values are oscillatory as the time goes on, in response to the pain-process during LBP.

**Fig. 2:**
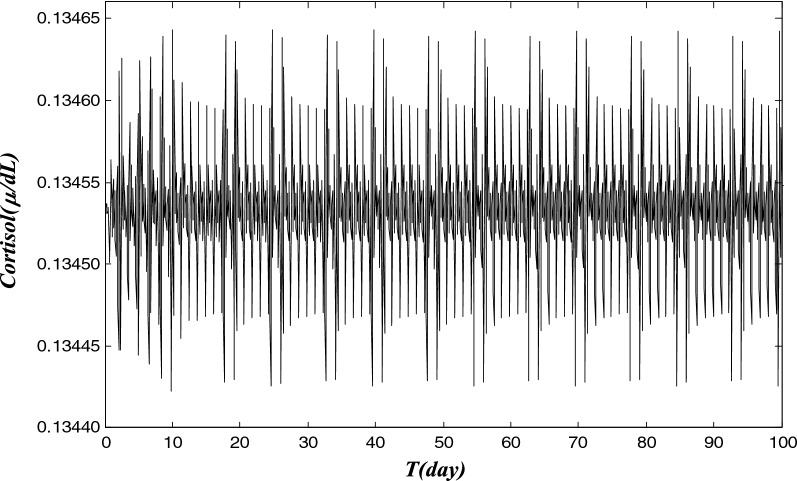
Simulated data of cortisol level during a period of 100 days, based on a HPA computational model that is described in Tables 1 and 3. The x-axis represents the time (unit: day) and the y-axis is the cortisol level. MATLAB solver ode45 (https://www.mathworks.com/help/matlab/ref/ode45.html) was used for its accuracy and relatively fast speed in computing this model. Due to the inherent structure of the HPA brain hormone system under stress, the cortisol level values are oscillatory as the time goes forward, in response to the pain-process during LBP. Cortisol data can be simulated for each hour, and each day contains 24-cortisol data points. This can be compared with clinical data in the future, where cortisol secretion of patients can be monitored in real time and provide further insight for LBP and pain trajectory patterns. The mathematical HPA model, and its biological meaning of the parameters, are provided in Tables 1, 2. A typical set of HPA parameters for LBP is given in Table 3

The time trajectory of cortisol-level is presented in Fig. 2, during the first 100 days for the HPA computational model. The cortisol values are given at each hour. Cortisol data can be simulated for any instance, but we only present the data at each hour, and each day contains 24-cortisol data points. Due to the inherent structure of the HPA brain hormone system under stress, the cortisol level values are oscillatory as the time goes forward, in response to the pain-process during LBP. The values are between 0.13440 and 0.13465 μ/*DL* for the entire times, and the up and down cycle is about 24 h. This simulated cortisol data can be compared with clinical data in the future for validation purpose, whereas cortisol secretion of patients can be monitored in real time and provide further insight for LBP and pain trajectory patterns.

**Fig. 3:**
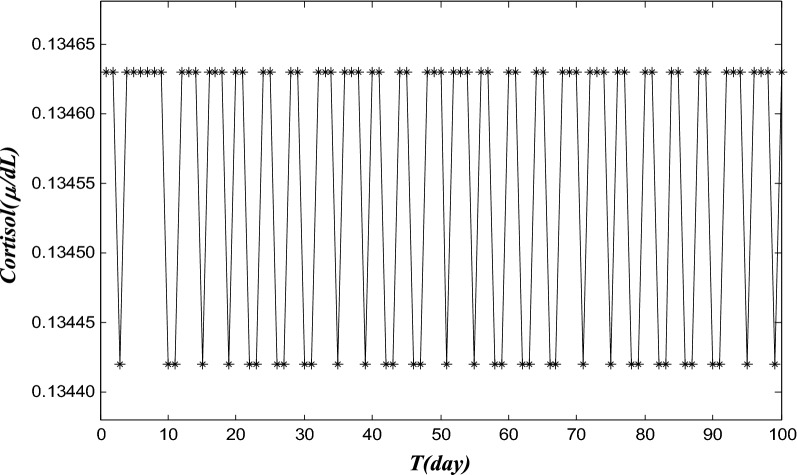
A simulated 2-state pain trajectory, based on the HPA modeling. The cortisol level values obtained from HPA model are classified into two different groups, and they present high-state and low-state patterns. One can use the higher cortisol level to model the high intensity pain, and the lower level to represent low-pain or no-pain. We first take a daily average of cortisol level (24 hourly data points). A subjective threshold (0.1345 in this model; around the average of extreme cortisol values) of cortisol level is used to separate the first 100 days into two groups: *high*-*pain day* (labeled in the high group as “cort = 0.13463”) for the days when cort 0.1345, or *low*-*pain day* when cort 0.1345 (labeled in the low group as “cort = 0.13442”) for trajectory study. The simulated pain trajectory is representative for a LBP episode that has chronic and intermittent pain

In Fig. 3, a simulated pain trajectory, based on HPA-modeling analysis, is presented.

The cortisol-level values were classified into two different groups, and they present high-state and low-state patterns. The higher-cortisol level was used to model the high-intensity pain, and the lower-level to represent the low-pain or no-pain. We took a daily average of cortisol values for the study period. Then, for the averaged cortisol values, a subjective threshold of cortisol level was taken to separate the daily pain states into two groups (high-pain or low-pain) for the trajectory study. The simulated pain trajectory in two states (high-pain and low-pain) is presented in Fig. 3. We first took a daily average of cortisol level (24 hourly cortisol data points). A subjective threshold (0.1345 in this model; around the average of extreme cortisol values) of cortisol level was used to separate the first 100 days into two groups: high-pain day for these days when cort ≫ 0.1345, or low-pain day when cort < 0.1345, for pain trajectory study. The simulated pain trajectory depicted in Fig. 3 is representative for a LBP episode that has chronic and intermittent pain.

The HPA-system parameters that reflect the synaptic connectivity are crucial in setting pain-states. One can also analyze the dynamics-pattern changes induced by any parameter variation, in order to understand LBP transition from acute to chronic pain. The HPA-system parameters reflect the synaptic coupling strength or neuron degradation, and they are crucial in setting pain-states. One can systematically analyze the dynamic pattern-changes induced by any parameter variation, in order to understand LBP transition from acute to chronic pain.

**Fig. 4:**
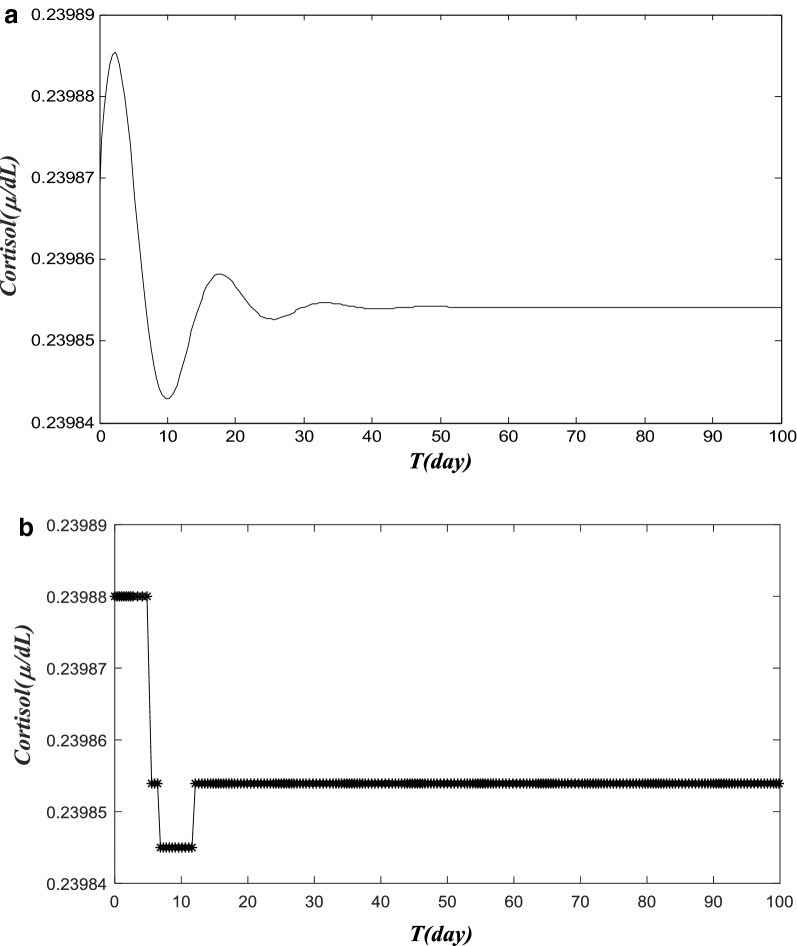
A simulated cortisol level data and pain trajectory for acute pain. **a** A simulated cortisol level of acute pain over the 100-day period. Here, we present a case of simulated cortisol dynamics that is with acute pain that quickly subsides in 1–2 weeks. We modify a HPA model with a reduced adreno-corticotrophin hormone degradation rate (Vs4 is reduced from 15 to 0.907 µg days), while others remain at a normal level. The cortisol level quickly returns to a flat level in** a**, after a few initial oscillations. **b** Simulated 3-state pain trajectory calculated from simulated cortisol data in **a**. The cortisol level values obtained from HPA model are classified into three different groups, and they present high pain state, low pain state and no pain (healed) state patterns. Again, we first take a daily average of cortisol level (24 hourly data points). Subjective thresholds (0.23986 and 0.2985 in this model) of cortisol level are used to separate the first 100 days into three groups: high-pain day (labeled in the high-pain group) for the days when cort ≫ 0. 23986, or healed day when 0.23986 > cort ≫ 0. 23985 (labeled in the healed or no-pain group), and low-pain for cort < 0. 23985 for trajectory studies. The simulated pain trajectory is representative for an acute LBP episode and the corresponding pain trajectory will converge quickly to the healed or no-pain state

In Fig. 4a, b, a case of cortisol dynamics of acute pain was simulated over a 100-day period, with acute pain that quickly subsides in 1–2 weeks, whereas a HPA with a reduced adreno-corticotrophin hormone degradation was considered (Vs4 was reduced from 15 µg d to 0.907 µg d, while others remain at normal level). Figure 4a depicts the cortisol level in the 100 days. The cortisol level quickly returns to a flat level in Fig. 4a, after a few initial oscillations. In Fig. 4b, we presented a 3-state pain trajectory calculated from simulated cortisol data in Fig. 4a. Similarly, we first took a daily average of cortisol level (24 hourly data points). Subjective thresholds (0.23986 and 0.2985 in this model) of cortisol level are used to separate the days into three groups: high-pain day (labeled in the high-pain group on the top) for the days when cort ≫ 0. 23986, or healed day when 0.23986 > cort ≫ 0. 23985 (labeled in the healed or no-pain group in the middle), and low-pain for cort < 0. 23985, for trajectory studies. The simulated pain trajectory is representative for an acute LBP episode, and the corresponding pain trajectory converges quickly to the healed or no-pain state, as shown in Fig. 4b.

**Fig. 5:**
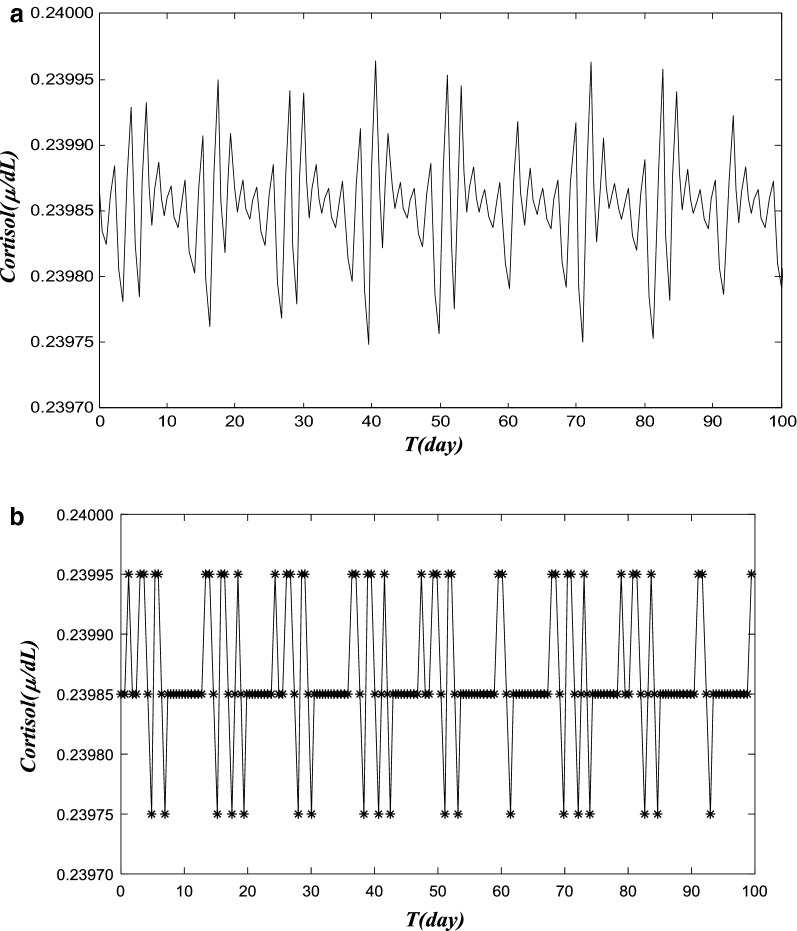
The cortisol trajectory data for a manic-style pain. **a** A simulated cortisol level of manic-style pain over the 100-day period. This is simulated with a patient who can quickly recover from acute pain with identical parameters as in Fig. 3, when the stress is elevated (K-stress is increased from 10.1 to 30 µg days). The cortisol trajectory can transition from acute pain to a “manic” oscillatory pattern, based on simulated cortisol level data for the HPA model with the new parameter set. The cortisol level is presented here. The nearly periodic patterns have a period around 10, and alternate between high-amplitude oscillations and low-amplitude ones during the 100 days time period. **b** Pain trajectory calculated from data in **a** for 100 days of the HPA model simulation. The cortisol level values obtained from HPA model are classified into three different groups, and they present high pain state, low pain state and no pain (healed) state patterns. We take a daily average of cortisol level (24 hourly data points). Subjective thresholds (0.23990 and 0.29980 in this model) of cortisol level are used to separate the first 100 days into three groups: high-pain day (labeled in the high-pain group in the top) for the days when cort ≫ 0. 23990, or healed day when 0.23990 > cort ≫ 0.23980 (labeled in the healed or no-pain group in the middle), and low-pain for cort < 0.23980 (in the bottom) for trajectory studies. The simulated pain trajectory is representative for a *manic*-*style pain* LBP

Then, in Fig. 5, an HPA-system of “manic” pain was simulated, when the stress is elevated (Kstress is increased from 10.1 to 30 µg days), with other parameters identical as in Fig. 4; the cortisol trajectory transforms into a “manic” oscillatory pattern, as shown in Fig. 5a. The nearly periodic patterns have a period around 10, and alternate between high-amplitude oscillations and low-amplitude ones during the 100 day time period. In Fig. 5b, pain trajectory calculated from data in Fig. 5a shows a similar manic pattern. The cortisol level values obtained from HPA model are classified into three different groups, and they present high pain state, low pain state and no pain (healed) state patterns. We first took a daily average of cortisol level (24 hourly data points). Subjective thresholds (0.23990 and 0.29980 in this model) of cortisol level were used to separate the first 100 days into three groups: high-pain day (labeled in the high-pain group in the top) for the days when cort ≫ 0. 23990; or healed day when 0.23990 > cort ≫ 0. 23980 (labeled in the healed or no-pain group in the middle); and low-pain for cort < 0. 23980 (in the bottom) for trajectory studies. The simulated pain trajectory is representative for a *manic*-*style pain* LBP.

### Pain trajectory of patient groups

**Fig. 6:**
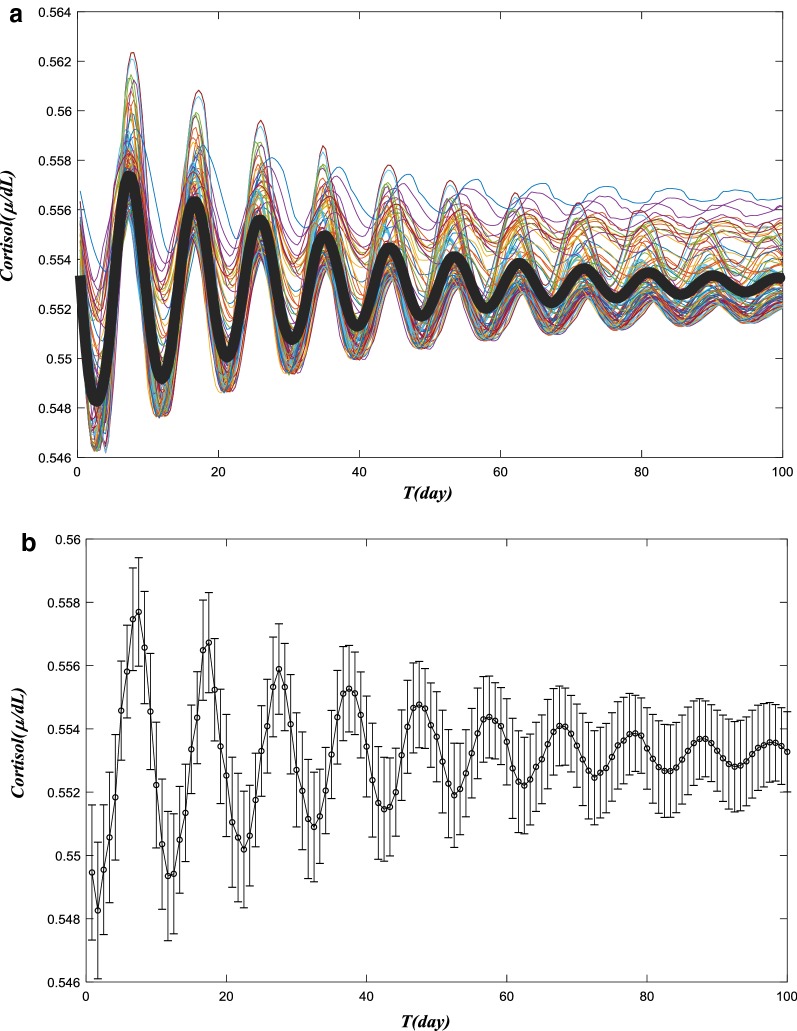

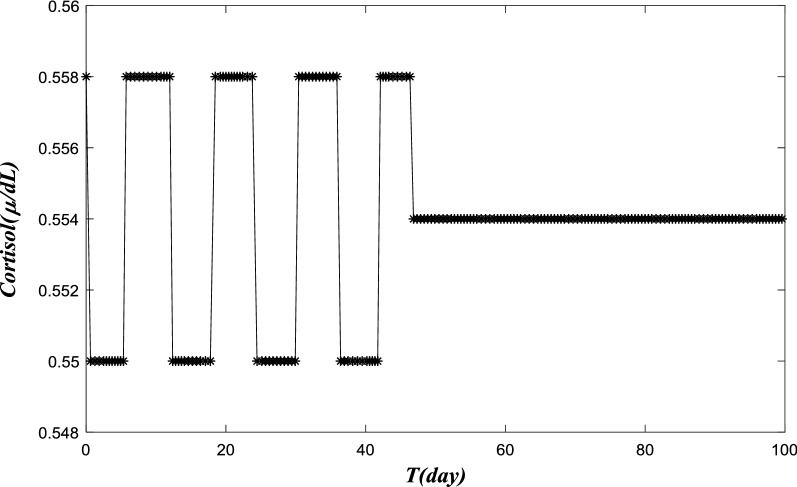
The cortisol trajectory and pain trajectory modeling for a group of 100 patients. **a** Simulated cortisol level for LBP by the HPA model in a group of 100 patients. We keep all same parameters in the HPA model as in Fig. 4 except Kstress, and we choose 100 randomized values for Kstress. The twenty values are randomly generated by computer, the average Kstress is 0.9922, and the variance is 0.2907. Then we simulated the HPA model for each Kstress. The cortisol trajectories vary among patients, from high-amplitude oscillations and low-amplitude ones during the 100-day time period. Each patient’s cortisol data is presented in **a** with different color. **b** Average cortisol level for LBP by the HPA model in a group of 100 patients. We also calculated the average daily cortisol-levels to be used to calculate the group-pain trajectory. The level variance is also marked in the group. For the group, the average and variance become stabilized after 60 days. **c** Pain trajectory calculated from data in Fig. 4a for 100 days of the patients group. The average cortisol level values obtained from HPA model are classified into three different groups, and they present high pain state, low pain state and no pain (healed) state patterns respectively. We take a daily average of cortisol level (24 hourly data points). Subjective thresholds (0.556 and 0.552 in this model) of cortisol level are used to separate the first 100 days into three groups: high-pain day (labeled in the high-pain group in the top) for the days when cort ≫ 0.556, or healed day when 0.556 > cort ≫ 0.552 (labeled in the healed or no-pain group in the middle), and low-pain for cort < 0.552 (in the bottom) for trajectory studies. The simulated pain trajectory is representative for a group of LBP patients who recovered from acute pain in various severities

We now a present a computer-simulated study of pain trajectories in a group of patients. Unlike an individual patient whose hormonal levels are uniquely decided, we expect a distribution of cortisol levels among different patients. We assume the cortisol dynamics values are normalized (as in [57], so the discrepancy is due to random fluctuation or variation among individual patients. We chose a sample of 100 random values of a reduced adreno-corticotrophin hormone degradation rate (Vs4) and the stress coefficient (Kstress). We first evaluated the cortisol dynamics, and took the averaged values of 100 patients. Then, we developed the pain trajectory for this group of patients. We also calculated the variance of the cortisol-level and pain-trajectories. Figure 6a and b depict the average dynamics and their variances in cortisol level during the first 100 days, We kept all the same parameters in the HPA model as in Fig. 4, except Kstress, and we choose 100 randomized values for Kstress. The 100 values were randomly generated by the computer; the average Kstress is 0.9922, and the variance is 0.2907. Then, we simulated the HPA model for each Kstress. The cortisol trajectories vary among patients, from high-amplitude oscillations and low-amplitude ones during the 100-day time period. Each patient’s cortisol data is presented in a, with different color as shown in Fig. 6a. We also calculated the average daily cortisol-levels to be used to calculate the group-pain trajectory. The level variance is also marked in the group. Both daily values are presented in b. For the group, the average and variance become stabilized after 60 days. The average cortisol level values were then used to obtain a 3-state pain trajectory. We took a daily average of cortisol level (24 hourly data points). Subjective thresholds (0.556 and 0.552 in this model) of cortisol level were used to separate the first 100 days into three groups: high-pain day (labeled in the high-pain group in the top) for the days when cort ≫ 0.556; or healed day when 0.556 > cort ≫ 0.552 (labeled in the healed or no-pain group in the middle); and low-pain for cort < 0.552 (in the bottom) for trajectory studies. Shown in Fig. 6c, the simulated pain trajectory is representative for a group of LBP patients who recovered from acute pain in various severities.

## Discussion

The simulations revealed in the present study demonstrate the feasibility of studying pain trajectory and pain transition, based on cortisol dynamics of a HPA-computational model. From a representative cortisol-value change, we can construct a fairly good pain trajectory for an individual patient, as well as a group of patients. With variation of parameters representing synaptic connectivity and neural degrading, a cortisol dynamics and pain trajectory study can provide various scenarios of acute, manic-style pain and intermittent/chronical pain. However, one will need to “fine-tune” the parameters with other pain-related measures and biomarkers in the future. Our ultimate goal is to use computational-bifurcation analysis in order to predict the outcome of the initial pain trajectory, and then the transition to different types of trajectories (i.e., acute pain and chronic pain in LBP), based on stable-patterns in different parameter-sets. This mathematical tool has been very successful in predicting the pattern-transition during epileptic seizures in an extended Taylor model (Fang et al. in press), and breathing irregularity induced by the changes in Pre-Bőtzinger Complex (a pacemaker in deep-brain regions; [12, 15].

It has been established that interdisciplinary treatment strategies are generally most effective for pain management, allowing for the customization of a treatment plan for the individual patient [5]. Understanding how to identify pain trajectories would be of great importance in tailoring treatment-strategies to patients experiencing pain, both at the acute and chronic stages. Kongsted et al. [26] iterate that other than known factors, such as activity-limitation, work participation, history of back or leg pain, anxiety, and catastrophizing, there were also observed relationships between pain patients and mixed-evidence concerning sleep-disturbances and patient-education level. They further note that these variables were not used to group patients into trajectory patterns. It would be useful to determine if these associations will affect the trajectory a patient follows long-term. Our computational modeling is currently not sophisticated enough to include all these factors. Fortunately, there are currently available cutting-edge methods, such as daily electronic diaries using smart phones (e.g., [21, 23, 25, 32, 33] to systematically track patients from the acute LBP injury and over the following one-year period, using an inception-cohort study design. Finally, these methods will supplement the HPA-axis predictive data to further provide guidance on biomarkers that may be used to better understand the underlying neuroscientific-transition mechanisms involved in order to help prevent CLBP.

## Conclusions

Computational-modeling based on HPA-axis can also provide insight into the mechanisms of pain, and the pathways from acute injury to chronic conditions, and collaborate with pain trajectory patterns. There are, though, currently limitations of predictive modeling, as it primarily depends on HPA-hormonal dynamics only. However, illuminating pain trajectories and subsequently developing effective and customizable prevention and intervention techniques for the growing adult population is critical [16]. Many trajectory patterns have been proposed in the CLBP population, and we must develop a cohesive understanding of how and why these patterns are emerging in order to provide the best possible medical care for those suffering with pain. Promoting ideal outcomes, particularly in the aging population, requires all-inclusive comprehension of risk factors and triggers.

## Data Availability

All data generated or analyzed during this study are included in this published article.
